# 10-Dehydrogingerdione Attenuates Tramadol-Induced Nephrotoxicity by Modulating Renal Oxidative Stress, Inflammation and Apoptosis in Experimental Rats: Role of HO-1 Activation and TLR4/NF-κB/ERK Inhibition

**DOI:** 10.3390/ijms23031384

**Published:** 2022-01-26

**Authors:** Gehad M. Elnagar, Mohamed M. Elseweidy, Yasmin K. Mahmoud, Nesreen M. I. M. Elkomy, Ziyad M. Althafar, Sultan F. Alnomasy, Naif A. Al-Gabri, Mohamed Shawky

**Affiliations:** 1 Biochemistry Department, Faculty of Pharmacy, Zagazig University, Zagazig 44519, Egypt; dr.gehadelnagar@yahoo.com (G.M.E.); yasminkassem33@yahoo.com (Y.K.M.); 2 Pharmacology and Toxicology Department, Faculty of Pharmacy, Zagazig University, Zagazig 44519, Egypt; dr_nesreen55@yahoo.com; 3 Department of Medical Laboratories Sciences, College of Applied Medical Sciences in Al-Quwayiyah, Shaqra University, Riyadh 11564, Saudi Arabia; zaldosari@su.edu.sa (Z.M.A.); s.alnomasy@su.edu.sa (S.F.A.); 4 Department of Pathology, Faculty of Veterinary Medicine, Thamar University, Dhamar 124401, Yemen; naifaljabry@yahoo.com; 5 Laboratory of Pathology, Salam Veterinary Group, Buraydah 51911, Saudi Arabia; 6 Department of Biochemistry, Faculty of Pharmacy, Horus University, New Damietta 34711, Egypt; mohamedshawky1@gmail.com

**Keywords:** tramadol, nephrotoxicity, 10-dehydrogingerdione, heme oxygenase-1, toll-like receptor 4, NF-κB, Caspase-3

## Abstract

Tramadol represents a synthetic opioid analgesic especially for mild to severe pain. Its dose must be commonly monitored according to pain status and to alleviate the appearance of any adverse effects such as renal cellular damage during its excretion. Present work aimed mainly to study the effects of tramadol intake on renal tissues and 10-dehydrogingerdione (10-DHGD) potential as a protective agent. Tramadol administration induced an increase in serum levels of urea, creatinine, uric acid, the renal immune expression of nuclear factor kappa-light-chain-enhancer of activated B cells (NF-κB), and caspase-3 which turned out to be decreased by 10-DHGD intake. Our results also recorded a significant increase in renal malondialdehyde (MDA), toll-like receptor 4 (TLR4), and extracellular signal-regulated protein kinase-1 (ERK1) along with glutathione (GSH), superoxide dismutase (SOD), and heme oxygenase-1 (HO-1) decrease due to tramadol intake, which were counteracted by 10-DHGD administration as illustrated and supported by the histopathological findings. Our conclusion refers to renoprotective potential of 10-DHGD against tramadol adverse effects.

## 1. Introduction

Tramadol represents a synthetic opioid analgesic and is mainly used to alleviate mild to severe pain through complicated mechanism [1]. Previous studies indicate that tramadol and other opioid agents induce sedation and additional clinical effects [1,2]. Tramadol usually binds to μ-opioid receptors and to a lesser extent than morphine [1]. It also prevents nerve uptake of serotonin and norepinephrine similar to antidepressant effects of amitriptyline and desipramine [3]. It is metabolized in the liver and its biotransformation products are excreted by the kidneys [4]. Tramadol has a dose-dependent analgesic effect as compared to codeine and morphine [5]. Therefore, tramadol dose must be regulated according to the pain severity since the side effects arisen represents an earnest problem for patients and providers of health care [6].

Drug intake is usually associated with certain liver toxicity due to its involvementin drug metabolism [7], a pre-step for its renal excretion with subsequent induction of renal cellular damage [8]. It is believed that Tramadol is low in abuse and has no side effects, such as addictive drugs, while its overdose may lead to depression of central nervous system, respiration followed by vomiting, nausea, irregular heartbeat, coma, seizures, heart, and vascular collapse [9]. Long-term tramadol use to relieve pain and for those individuals seeking the drug is dialectical as its effects at the cellular level are not well understood [10].

Several studies indicated a link between tramadol use and the production of reactive oxygen species (ROS) [11]. The latter can induce significant damage to the cell components, formation of secondary toxic compounds [12], and subsequent apoptosis [13].

Heme oxygenase-1 (HO-1) is a microsomal cytoprotective enzyme that is induced in response to injury and cellular stress [14]. Importantly, HO reduces toxicity by degrading heme released from heme-containing proteins such as hemoglobin, myoglobin, and cytochromes, using nicotinamide adenine dinucleotide phosphate (NADPH) and molecular oxygen as cofactors [15]. The enzymatic mechanism has been elucidated, and structural studies provide insight into function [16], and bilirubin represents a free radical scavenger that blocks lipid peroxidation [17].

Toll-like receptors (TLRs) represent a signal transduction molecule that is involved in the induction of both innate and adaptive immunity. Recent evidence suggests that Toll-like receptor 4 (TLR4) is activated by endogenous proteins released from damaged tissues and plays a role in mediating renal injury following ischemia/reperfusion (I/R) [18]. TLR4 activation for renal parenchymal cells may thus activate mitogen-activated protein kinase (MAPK) pathways, resulting in increased production of inflammatory cytokines such as NF-κB and subsequent kidney injury [19]. As a result, targeting TLR4 signaling pathways may represent a therapeutic strategy to prevent tramadol-induced kidney injury.

10-Dehydrogingerdione (10-DHGD), a natural product derived from the rhizomes of *Zingiber officinale*, has remarkable antioxidant, anti-inflammatory, and nephroprotective properties [20]. The present study aimed mainly to illustrate the renal tissues injury induced in subsequent to tramadol intake and 10-DHGD potential to counteract nephropathy progression.

## 2. Results

### 2.1. Body Weight, Kidney Weight, and Kidney Weight to Body Weight Ratio

Nonsignificant differences were observed regarding body and kidney weights between tramadol-received rats and the control group. 10-DHGD alone and its combination with tramadol, showed also nonsignificant differences compared to control, tramadol, or 10-DHGD groups (Table 1).

### 2.2. Serum Kidney Function Parameters

Rats that received tramadol demonstrated significant increase in serum creatinine, uric acid, and urea (*p* < 0.001) as compared to control. Co-administration of 10-DHGD with tramadol attenuated such increase (*p* < 0.001) compared with the tramadol group (Table 2).

### 2.3. Renal Oxidative and Antioxidant Markers

Kidney tissue illustrated high oxidative stress as manifested by a significant increase in renal MDA levels along with significant decreases of SOD, GSH, and HO-1 levels compared with the control group (*p* < 0.001). 

Renal MDA and HO-1 levels showed nonsignificant difference in 10-DHGD or its combination with tramadol while renal SOD and GSH demonstrated significant decrease compared with control.

**Figure 1:**
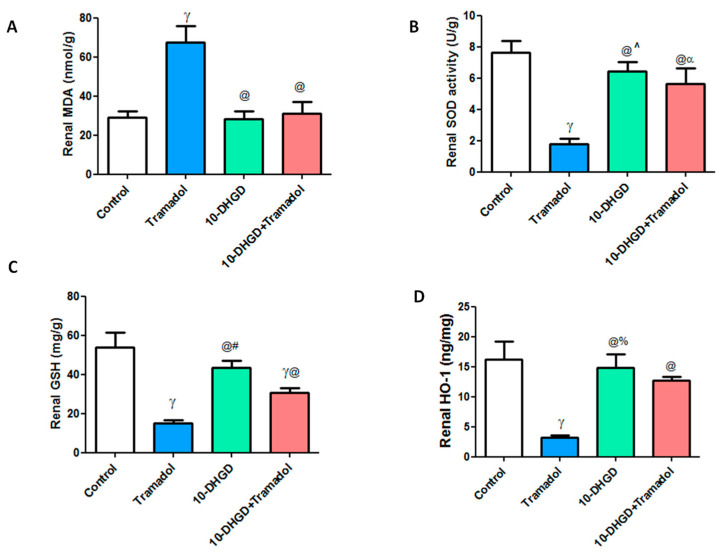
Effects of 10-DHGD administered either individually or combined with tramadol on renal oxidative and antioxidant markers in experimental rats for 45 days (**A**–**D**). Control: normal chow-fed rats received vehicles for 45 days; Tramadol: rats received tramadol HCl (20 mg/kg body weight) i.p. and daily for 45 days; 10-DHGD: rats received 10-dehydrogingerdione (10 mg/kg) orally and daily for 45 days; 10-DHGD + tramadol: rats received a combination of 10-DHGD and tramadol daily for 45 days; MDA: malondialdehyde; SOD: superoxide dismutase; GSH: glutathione; HO-1: heme oxygenase-1. Data expressed as mean ± SD, (*n* = 6/group); ^α^ *p* < 0.05, ^γ^ *p* < 0.001 vs. control; ^@^ *p* < 0.001 vs. tramadol; ^^^ *p* < 0.05, ^%^ *p* < 0.01, ^#^ *p* < 0.001 vs. 10-DHGD + tramadol.

10-DHGD administration either individually or in combination with tramadol significantly ameliorated renal injury as demonstrated by decreased oxidative stress markers and MDA, along with increased antioxidant parameters, SOD, GSH, and HO-1 as compared to the tramadol group (*p* < 0.001) (Figure 1).

### 2.4. Renal Toll-like Receptor-4 and Extracellular Signal-Regulated Protein Kinase-1

TLR4 is an important mediator of inflammation leading to renal injury where it is significantly increased in the tramadol group as compared to control (*p <* 0.001). 10-DHGD administered either individually or combined with tramadol greatly decreased levels of TLR4 by 89.8% and 85.9%, respectively, compared with the tramadol group. The effect of 10-DHGD alone was higher than its combination with tramadol by 31.2% (*p <* 0.05).

**Figure 2:**
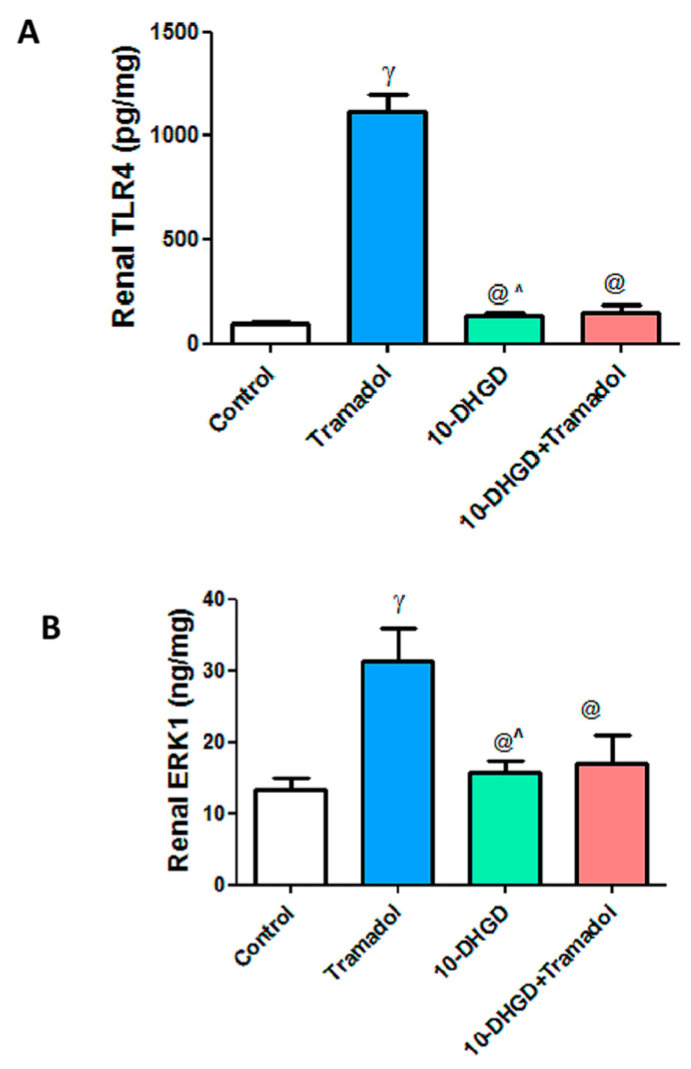
Effects of 10-DHGD administered either individually or combined with tramadol on renal Toll-Like Receptor-4 and extracellular signal-regulated protein Kinase-1 in experimental rats for 45 days (**A**,**B**). Control: normal chow-fed rats received vehicles; tramadol: rats received tramadol HCl (20 mg/kg body weight) i.p. and daily for 45 days; 10-DHGD: rats received 10-dehydrogingerdione (10 mg/kg) orally and daily for 45 days; 10-DHGD+tramadol: rats received a combination of 10-DHGD and tramadol daily for 45 days; TLR4: toll-like receptor-4; ERK1: extracellular signal-regulated protein kinase-1. Data expressed as mean ± SD, (*n* = 6/group); ^γ^ *p* < 0.001 vs. control; ^@^ *p* < 0.001 vs. tramadol; ^^^ *p* < 0.05 vs. 10-DHGD+tramadol.

Similarly, ERK1 recorded significant increase in the tramadol group by 2.4-fold as compared to healthy ones (*p <* 0.001). Individual or combined administration of 10-DHGD significantly decreased ERK1 level by 55.4% & 43.5%, respectively, compared with tramadol alone (*p* < 0.001), (Figure 2).

10-DHGD alone or combined with tramadol demonstrated nonsignificant difference concerning both parameters in comparison with the control group.

### 2.5. Renal Histology

As shown in Table 3, tramadol induced significant alterations in histology pattern with multifocal inflammation, mild to moderate necrosis, diffuse hemorrhage focal fibrosis, and edema compared with the control group. Rats treated with 10-DHGD induced a significant improvement in renal histological lesions scores with still minimal of hemorrhages and glomerular alterations (thickened of glomerular membrane) as compared to the tramadol group. 

**Figure 3:**
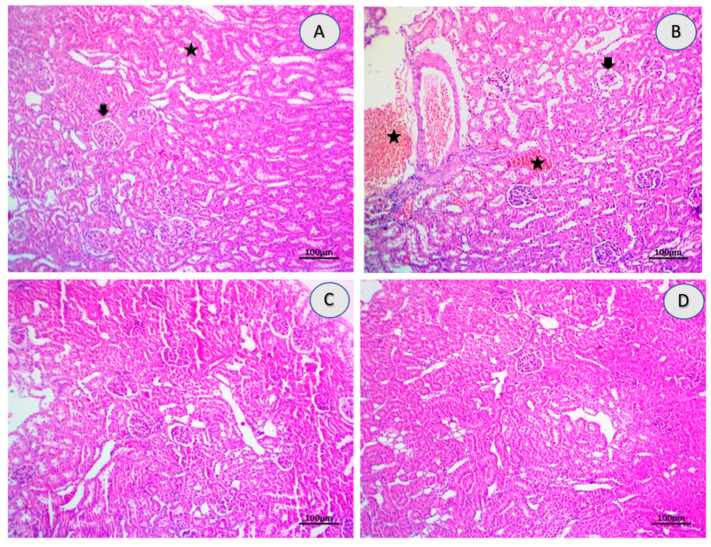
Photomicrographs showing H&E-stained renal sections (×100) and the effect of tramadol on kidney tissues. H&E-stained renal sections showed normal histomorphology structures including glomeruli (arrow) and renal tubules (star) in the control group (**A**), severe to moderate necrotic glomeruli (arrow), interstitial nephrites due to inflammatory cells infiltrations besides severe multifocal interstitial hemorrhagic (stars) in the tramadol group (**B**), normal renal glomeruli and proximal renal tubules in 10-DHGD (**C**), restoration of the histomorphology structures of the most cortical structures include glomeruli and proximal tubules and corticomedullary junctions in 10-DHGD+tramadol (**D**). Scale bar 100 μm.

**Figure 4:**
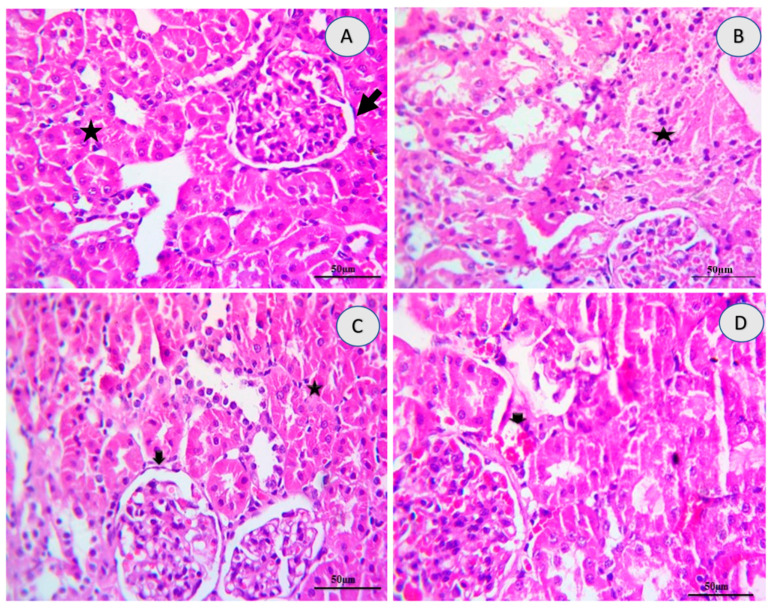
Photomicrographs showing high power H&E-stained renal sections (×400) and effects of tramadol on kidney tissues changes. Examined renal sections revealed normal glomerular tufts surrounded by normal basement membrane (arrow), apparently normal proximal renal tubules epithelium (star) in the control group (**A**), severe necrotic renal parenchyma infiltrated with lymphocytes (star), lobulated and congested glomeruli (arrow) in the tramadol group (**B**), normal glomerular tufts surrounded by normal basement membrane (arrow), apparently normal proximal renal epithelium of tubules (star) in 10-DHGD (**C**), apparently normal tubules and glomeruli with still minimal thickened basement membrane, and still little intact erythrocytes interstitially in 10-DHGD+tramadol (**D**). Scale bar 50 μm.

The tramadol group also revealed severe necrosis of renal parenchyma where the glomeruli are associated with interstitial nephritis due to inflammatory cells infiltrations, mainly lymphocytes admixed with necrotic debris, and severe multifocal interstitial hemorrhages. Moreover, renal glomeruli, distal and proximal renal tubules, in groups that received either 10-DHGD alone or in combination with tramadol, exhibited thickened basement membrane of a little glomeruli and intact erythrocytes interstitially (Figure 3 and Figure 4).

### 2.6. Renal NF-κB

**Figure 5:**
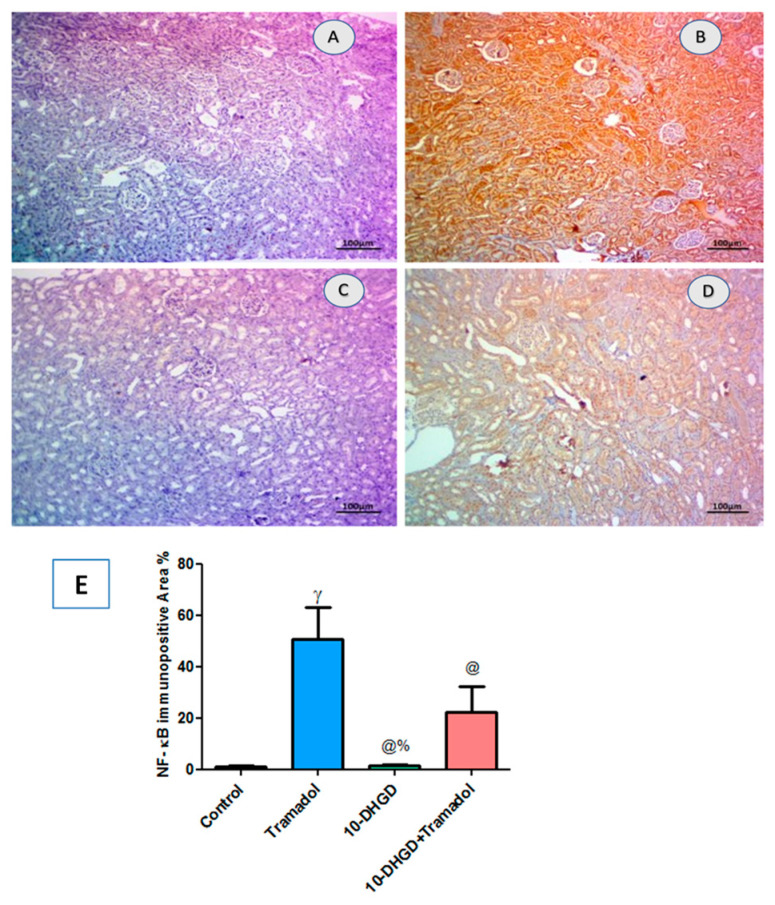
Photomicrographs showing NF-κB IHC-stained renal sections of cellular NF-κB and immunopositively execrations in renal sections of experimental rats. IHC-stained renal sections showed nearby normal expression in control group (**A**) and extensive immunoexpression in the tramadol group (**B**) which was significantly decreased in 10-DHGD (**C**) while moderately declined in 10-DHGD+tramadol (**D**). Scale bar 100 μm. For quantifying analysis, the expression of such parameter was determined as the mean area percentage of immunopositivity cells (**E**). Five microscopic fields were inspected, and an average value was calculated for each animal. Three renal sectors from 3 different rats were used for each group. (**E**) Vertical columns represent mean, while error bars represent standard deviation.IHC: immunohistochemical; NF-κB: nuclear factor kappa-light-chain-enhancer of activated B cells; control: rats fed a normal rodent chow diet and vehicles for 45 days; tramadol: rats received tramadol (20 mg/kg body weight) i.p. for 45 days;10-DHGD: rats received 10-DHGD (10 mg/kg body weight/day) for 45 days; 10-DHGD+tramadol: rats received 10-DHGD and tramadol for 45 days. ^γ^ *p* < 0.001 vs. control; ^@^ *p* < 0.001 vs. tramadol; ^%^ *p* < 0.01 vs. 10-DHGD+tramadol.

As shown in Figure 5, the tramadol group displayed extensive immune expression of NF-κB in renal tissues compared with the control group (*p <* 0.001) and significantly decreased in the 10-DHGD group along with moderate decline in 10-DHGD combined with tramadol as compared to tramadol alone (*p <* 0.001). 10-DHGD demonstrated a significant decline as compared to 10-DHGD+tramadol (*p <* 0.001).

### 2.7. Renal Apoptotic Marker (Caspase-3)

**Figure 6:**
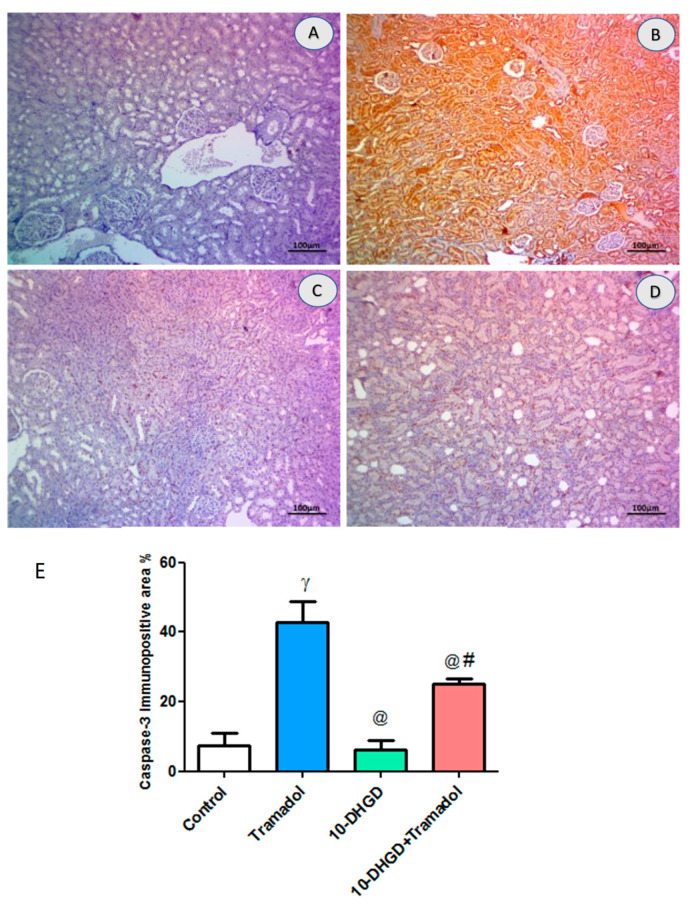
Photomicrographs showing Caspase-3 IHC-stained renal section. IHC-stained renal sections showed nearby normal expression in control group (**A**) and extensive immune expression in the tramadol group (**B**), which turned to be decreased significantly in the 10-DHGD group (**C**), while moderate decline was seen in the combined group (**D**). Scale bar 100 μm. For quantifying analysis, the expression of such parameter was determined as the mean area percentage of immunopositivity cells (**E**). Five microscopic fields were inspected, and an average value was calculated for each animal. Three renal sectors from 3 different rats were used for each group. (**E**) Verticalcolumns represent mean, while error bars represent standard deviation. IHC: immunohistochemical study; control: rats fed a normal rodent chow diet and vehicles for 45 days; tramadol: rats received tramadol (20 mg/kg body weight) i.p. for 45 days; 10-DHGD: rats received 10-DHGD (10 mg/kg body weight/day) for 45 days; 10-DHGD + tramadol: rats received 10-DHGD and tramadol for 45 days. ^γ^ *p* < 0.001 vs. control; ^@^ *p* < 0.001 vs. tramadol; ^#^ *p* < 0.01 vs. 10-DHGD+tramadol.

Immunoexpression of Caspase-3 showed a significant increase in renal tissues of the tramadol group compared with control (*p <* 0.001); additionally, 10-DHGD administration ameliorated such increase compared with the tramadol group and 10-DHGD +tramadol (*p <* 0.001) (Figure 6).

## 3. Discussion

As mentioned before, tramadol hydrochloride is a synthetic opioid derivative having central effect [1,21,22] and usually induce oxidative stress.

The latter represents an imbalance state between cellular ROS generated and subsequent release of antioxidant enzymes inducing pathological consequences [17,23]. ROS generally controls many signaling pathways, including cell growth, differentiation, mitogenesis, extracellular matrix (ECM) formation, degradation, inflammation, and apoptosis [23]. Oxidative stress is also a mediator for cellular damage, which is mainly counteracted by antioxidant defense systems such as heme oxygenase-1 (HO-1) along with lipid peroxidation inhibition [17]. Present results indicated that tramadol administration significantly decreased the antioxidant effect of renal HO-1 and reduced glutathione (GSH) and superoxide dismutase (SOD), along with an increase of renal malondialdehyde (MDA) compared with the control group; meanwhile, 10-DHGD exhibited renal protective effect.

Toll-like receptor 4 (TLR4) is a classical pathogen recognition receptor which plays a critical role in the innate immune system [24,25] and can activate nuclear factor kappa-B (NF-κB) [26]. TLR4 is triggered by pathogen-associated substances, which may activate the NF-κB signaling pathway leading to the production of proinflammatory cytokines and chemoattractants. The transcription factor NF-κB is a critical component of the inflammatory response, and its activated form is transported to the nucleus, where it induces the transcription of target genes such as proinflammatory cytokines in tubular cells and infiltrating immune cells [26].

Excessive ROS can induce kidney damage by activating protein kinase C, mitogen-activated protein kinase (MAPK), transcription factors, and cytokines [27]. ROS can also activate NF-κB and p38 MAPK which, in turn, activate NLRP3 inflammatory bodies [28]. The activation of extracellular signal-regulated kinase (ERK) 1/2, c-Jun N-terminal kinase (JNK) 1/2/3, and p38 MAPK signaling pathways are all involved in MAPK signaling. MAPK and ERK have a role in cell proliferation, differentiation, apoptosis, and inflammation [29].

Tramadol intake increased renal TLR-4 followed by significant increase in renal NF-κB compared with the control group. 10-DHGD administration, either individually or in combination with tramadol, induced significant decrease of these parameters as compared to the tramadol group. 

Accordingly, the activation of NF-κB can induce concomitant activation of apoptosis signaling, such as renal ERK-1 and caspase-3 expression, in the tramadol group compared with control. 10-DHGD administration in combination with tramadol significantly decreased ERK-1 and renal expression of caspase-3 compared with tramadol alone, exerting its potential as a protective agent against renal injury.

Histopathological results have offered a great support to our biochemical data. This is illustrated as severe to moderate necrotic glomeruli and interstitial nephritis due to inflammatory cells infiltrations in tramadol group. Severe multifocal interstitial hemorrhagic that were also observed turned out to be alleviated to a greater extent in the group that received 10-DHGD.

## 4. Materials and Methods

### 4.1. Animals

Twenty-four adult male albino rats weighing 150 ± 10 g were provided by the Faculty of Veterinary Medicine, Zagazig University (Egypt). The animals were kept in plastic cages with woods have bedding at the animal care facility of Zagazig University’s Faculty of Pharmacy, on a 12 h light/dark cycle, with free access to food and tap water. The temperature and humidity of the animal housing were kept constant (temperature 23 ± 2 °C, humidity 60% ± 10%). Prior to the experiment, the rats were acclimatized for two weeks and fed a commercially normal chow diet (El Nasr Company for Pharmaceutical and Chemicals, Zagazig, Egypt). All the procedures were approved by Institutional Animal Care and Usage Committee (IACUC) of Zagazig University (ZU) (Protocol 2018_F_3, permission code, 7 April 2018). 

### 4.2. Drugs and Chemicals

Tramadol hydrochloride (≥99%) was kindly supplied from the Forensic Medicine Department, MPH, Cairo, Egypt. 10-DHGD was extracted from ginger fresh rhizomes (*Zingiber officinale*), identified, and purified as described previously [30]. All chemicals used in this study were of analytical grade. 

### 4.3. Experimental Design

Initial body weights of rats were determined first, then they were randomly distributed into four groups (six of each) as the following: 

Group 1 (Control): Rats received a regular normal chow diet and tap water together with vehicles (2% gum acacia by oral gavage and physiological saline intraperitoneal for 45 days).

Group 2 (Tramadol): Rats received tramadol HCl (20 mg/kg body weight), dissolved in physiological saline (IP) and daily for 45 days.

Group 3 (10-DHGD): Rats received 10-DHGD (10 mg/kg body weight/day) for 45 days orally by gavage, suspended in water containing 2% gum acacia as suspending agent.

Group 4 (10-DHGD + Tramadol): Rats received 10-DHGD and were left for about one hour, then tramadol HCl was given IP using the same doses as mentioned above for 45 days. This is to alleviate to certain extent any kind of physical incompatibility.

### 4.4. Sampling

At the study end (45 days), rats were individually weighed, blood samples were collected *via* retroorbital sinus, and the blood samples were left to clot for 15 min at room temperature and then centrifuged at 4000 rpm for 15 min. Sera were separated and stored at −20 °C for further biochemical analyses. The animals were subsequently terminated using urethane anesthesia (1.2 g/kg, i.p.) and cervical dislocation [31]. Both kidneys were perfused then washed several times with sterile physiological saline (ice-cooled) to remove any contaminants and blotted dry using clean tissue paper. Right kidneys were sliced into four equal slices on icepacks, snap frozen in liquid nitrogen, and kept at −80 °C for further analysis. The left kidney was preserved in 10% neutral buffered formaldehyde and processed later for histopathological examination.

### 4.5. Biochemical Measurements

Serum creatinine and urea levels were measured colorimetrically using Diamonddiagnostic kits (Cairo, Egypt). Serum uric acid level was determined by enzymatic method using a Spinreact kit (Girona, Spain). Renal contents of malondialdehyde (MDA), glutathione (GSH), and superoxide dismutase (SOD) activity were performed using kidney tissue homogenates and laboratory biodiagnostics kits provided by Biotechnology CO, Giza, Egypt.

### 4.6. Enzyme Linked Immunosorbent Assay (ELISA) 

Heme oxygenase-1 (HO-1) activity in kidney tissue homogenates was measured using Rat HemeOxygenase-1 ELISA kit supplied by (MyBiosource Inc., San Diego, CA, USA) catalog no (MBS764989). Renal levels of toll-like receptor-4 (TLR-4) were measured using Rat Toll-Like Receptor 4 ELISA kit supplied by (MyBiosource Inc., San Diego, CA, USA) catalog no (MBS705488). Renal extracellular signal regulated kinase 1 (ERK 1) was measured using Rat ERK1 ELISA kit supplied by (MyBiosource Inc., San Diego, CA, USA) catalog no (MBS456854.We followed exactly the recommendation and instructions of the manufacturer’s 

### 4.7. Histopathological Examinations

Kidney tissues were properly fixed in 10% buffered neutral formalin solution for 48 h, then dehydrated in 70%, 90%, and 100% ascending grades of ethyl alcohol, and cleared in xylol and then embedded in paraffin wax. Five-micron thicknesses of paraffin were sliced with using a semi-automated microtome (Leica RM 2155, London, UK). The sections were prepared and then routinely stained with hematoxylin and eosin (H&E) for histopathology evaluations [32]. All section photos were photographed using a Leica^®^ microscope combined with AmScope^®^ microscope digital camera. Lesions score system was evaluated as the following: (0 = no detectable histopathological alterations, 1 = rarely minimal or focal, 2 = multifocal, 3 = patchy or diffuse) with a semiquantitative method [33].

### 4.8. Immunohistochemistry Analysis

Immunohistochemical staining for Caspase-3 and NF-κB proteins was performed in renal tissues according to the Avidin Biotin Complex technique described by Hsu et al., 1981 [34] using primary antibody (Abcam^®^). Briefly, 3–5micron-thick paraffin-embedded tissue slices were deparaffinized in xylene and subsequently rehydrated in decreasing HPLC alcohol. To avoid nonspecific binding sites, the sections were incubated in 5 percent bovine serum albumin (BSA) in Tris-buffered saline (TBS) for 2 h. The sections were incubated with primary antibody (Rabbit polyclonal IgG to rat Caspase-3 (Abcam^®^) at a concentration of 1g/mL containing 5% BSA in TBS and incubated overnight at 4 °C. After incubation, the slides were washed three times in TBS and then incubated for one hour at room temperature with goat anti-rabbit secondary antibody. TBS was used to wash the sections before incubating them in 0.02 percent diaminobenzidine (DAB) containing 0.01 percent hydrogen peroxide for 5–10 min. Hematoxylin was used to counterstain the sections, and the slides were examined under a microscope. Calculation of the percentage of immune stained areas in 10 fields of each slides were measured by using FijiImagJ^®^ software according to Rizzardiet al. [35].

### 4.9. Statistical Analysis

GraphPad Prism program version 5 was used for statistical analysis (Graph Pad Software, Inc., California, USA). One-way analysis of variance (ANOVA) and Tukey’s post hoc test were used to determine the statistical significance of differences between groups. A significant difference was expressed as *p* < 0.05.

## 5. Conclusions

10-DHGD has proven its antioxidant, anti-inflammatory, and anti-apoptotic properties because it alleviated, to a certain extent, the renal damage induced due to tramadol. 

## Figures and Tables

**Table 1:** Effect of 10-DHGD administration for 45 days either individually or combined with tramadol on body weight, kidney weight, and kidney weight to body weight ratio in experimental rats; data expressed as mean ± SD, (*n* = 6/group).

Groups	Initial Body Weight (g)	Final Body Weight (g)	Change in Body Weight	Kidney Weight (mg)	Kidney Weight to Body Weight Ratio (%)
Control	149.8 ± 7.59	200.9 ± 14.5	51.1	1.410 ± 0.17	0.67 ± 0.097
Tramadol	159.2 ± 6.76	227.4 ± 13.09	68.2	1.357 ± 0.13	0.62 ± 0.071
10-DHGD	151.8 ± 6.49	211 ± 12.94	59.2	1.502 ± 0.11	0.710 ± 0.07
10-DHGD+Tramadol	161.4 ± 6.35	222.6 ± 11.65	61.2	1.560 ± 0.14	0.715 ± 0.085

Control: normal chow-fed rats received vehicles for 45 days, tramadol: rats received tramadol HCl (20 mg/kg body weight) i.p. and daily for 45 days, 10-DHGD: rats received 10-dehydrogingerdione (10 mg/kg) orally and daily for 45 days, 10-DHGD+tramadol: rats received a combination of 10-DHGD and tramadol daily for 45 days.

**Table 2:** Effect of 10-DHGD administration for 45 days either individually or combined with tramadol on kidney function parameters in experimental rats.Data expressed as mean ± SD (*n* = 6/group).

Parameters	Control	Tramadol	10-DHGD	10-DHGD+Tramadol
Creatinine (mg/dL)	0.713 ± 0.053	1.453 ± 0.252 ^γ^	0.733 ± 0.040 ^@^^	0.993 ± 0.0637 ^@^
Urea (mg/dL)	46.67 ± 3.251	61.33 ± 5.49 ^γ^	50.00 ± 1.23 ^@^	53.00 ± 3.240 ^©^
Uric acid (mg/dL)	1.138 ± 0.121	4.263 ± 1.207 ^γ^	1.673 ± 0.234 ^@^	2.187 ± 0.297 ^@^

Control: normal chow-fed rats received vehicles for 45 days; tramadol: rats received tramadol HCl (20 mg/kg body weight)i.p. and daily for 45 days, 10-DHGD: rats received 10-dehydrogingerdione (10 mg/kg) orally and daily for 45 days, 10-DHGD+tramadol: rats received a combination of 10-DHGD and tramadol daily for 45 days. ^γ^ *p* < 0.001 vs. control; ^©^ *p* < 0.01, ^@^ *p* < 0.001 vs. tramadol; ^^^ *p* < 0.05 vs. 10-DHGD+tramadol.

**Table 3:** Lesions score of the severity extent in the renal tissues.

Lesions	Control	10-DHGD	Tramadol	Tramadol+10-DHGD
Inflammation (nephritis)	0	0	2	0
Necrosis/Degeneration	0	0	3	1
Hemorrhages	0	0	3	1
Cystic Dilatation	0	0	1	0
Fibrosis	0	0	1	0
Edema	0	0	2	0
Tubular Casts	0	0	3	0

Control: normal chow-fed rats received vehicles, tramadol: rats received tramadol HCl (20 mg/kg body weight) i.p. and daily for 45 days, 10-DHGD: rats received 10-dehydrogingerdione (10 mg/kg) orally and daily for 45 days, 10-DHGD+tramadol: rats received a combination of 10-DHGD and tramadol daily for 45 days. Lesions score system was as follows: (0 = no detectable histopathological lesion, 1 = rarely minimal or focal, 2 = multifocal, 3 = patchy or diffuse) as a semiquantitative method.

## Data Availability

The datasets generated during and/or analyzed during the current study are available from the corresponding author on reasonable request.
